# Elucidating the Inhibitory Potential of Statins Against Oncogenic c-Met Tyrosine Kinase Through Computational and Cell-based Studies

**DOI:** 10.5812/ijpr-158845

**Published:** 2025-08-16

**Authors:** Elham Ahmad Alizadeh, Leila Karami, Fahimeh Ghasemi, Amir Shadboorestan, Mohammad Reza Torabi, Vahideh Montazeri, Shima Aliebrahimi, Seyed Nasser Ostad

**Affiliations:** 1 Department of Pharmacy, Eastern Mediterranean University, Famagusta, North Cyprus; 2 Department of Cell and Molecular Biology, Faculty of Biological Sciences, Kharazmi University, Tehran, Iran; 3 Department of Medical Biotechnology, School of Advanced Technologies in Medicine, Tehran University of Medical Sciences, Tehran, Iran; 4 Department of Toxicology, Faculty of Medical Sciences, Tarbiat Modares University, Tehran, Iran; 5 Artificial Intelligence in Medical Sciences Research Center, Smart University of Medical Sciences, Tehran, Iran; 6 Department of Artificial Intelligence, Smart University of Medical Sciences, Tehran, Iran; 7 Department of Toxicology and Pharmacology, Toxicology and Poisoning Research Center, Faculty of Pharmacy, Tehran University of Medical Sciences, Tehran, Iran

**Keywords:** Boosting Machine Learning Algorithms, Molecular Docking Simulation, Molecular Dynamics Simulation, Stomach Neoplasms, Proto-oncogene Proteins c-Met, Hydroxymethylglutaryl-CoA Reductase Inhibitors

## Abstract

**Background:**

The cellular mesenchymal-epithelial transition (c-Met) receptor, a member of the receptor tyrosine kinase family, is a novel therapeutic target for treating many cancers, including stomach cancer. Overexpression of c-Met and/or high levels of hepatocyte growth factor (HGF) correlate with poor prognosis. Statins, as LDL-lowering agents, are exploited to obtain anti-cancer effects via a wide range of pleiotropic effects.

**Objectives:**

The present study aimed to discover the most effective statin as a c-Met signaling inhibitor through computational and experimental approaches.

**Methods:**

Two main computational approaches, i.e., machine learning (ML) model and molecular dynamics (MDs) simulation, were followed by cytotoxicity, flow cytometric analysis, and western blot assay on AGS and MKN-45 gastric cancer cells.

**Results:**

The machine learning section was founded on developing tree-based classification algorithms to predict the biological activities of the proposed statin structures as c-Met receptor inhibitors. In the second step, molecular docking and MD simulation were utilized to estimate the biomolecular interactions. The proposed classification models reveal that all structures have more than 200 nM biological activities. Machine learning led the experiment to find fluvastatin and pitavastatin as the two compounds with the highest inhibitory effects. In cell-based assays, both tested statins exhibited cytotoxicity and induced apoptosis, accompanied by sub-G1 accumulation in gastric cancer cells. However, no significant reduction in c-Met phosphorylation was observed by western blot.

**Conclusions:**

No relation between the statins’ inhibitory effect and the c-Met pathway on cancerous cells could be reported.

## 1. Background

The cellular mesenchymal-epithelial transition (c-Met) factor, also known as the hepatocyte growth factor (HGF) receptor, plays a crucial role in tumor development, including gastrointestinal cancers (1). Triggered by HGF, this signaling pathway is engaged in essential cellular processes such as growth, differentiation, metabolism, and apoptosis. However, its deregulation and the ensuing activity of downstream signaling cascades, including RAS-MAPK, PI3K-Akt, NF-κB, and Wnt/GSK-3β/β-catenin, contribute to cancerous progression, metastasis, and drug resistance (2, 3). Targeting the HGF/c-Met axis through small-molecule inhibitors is considered a promising therapeutic approach to hinder cancer progression (4, 5).

Gastric cancer is one of the most common cancer types globally (6). Standard treatment options include surgery, chemotherapy, and targeted therapy (7). Despite these multimodal therapies, the prognosis remains poor, leading to high mortality rates. Overexpression of the c-Met receptor has been attributed to poor prognosis in 22 - 82% of gastric cancers (8). Therefore, targeting the c-Met signaling pathway presents a potential strategy to improve gastric cancer treatment (9).

Statins, as HMG-CoA (HMGCR) agents, obtain anti-cancer effects possibly due to a wide range of pleiotropic effects contributing to treating neurological pathological conditions, inflammation, and even tumors (2). From a mechanistic perspective, HMGCR inhibition prevents mevalonate production, resulting in the blockage of isoprenoids such as geranylgeranyl pyrophosphate (GGPP1) and farnesyl pyrophosphate (FPP), which are responsible for post-translational modifications (prenylation) of G-protein subunits of RAS, RHO, RAB, RAC, and RAP. The aforementioned subunits are involved in cell membrane integrity, apoptosis, phagocytosis, vascular trafficking, and protein synthesis (10). Additionally, increased cholesterol synthesis and fatty acid metabolism have been attributed to tumor progression, and statin therapy may counteract this by inhibiting aberrant cell division (11). Besides, the purine-mimicking structure of small molecules, including statins, may directly block the ATP-binding site of the c-Met receptor (3, 4). Lipophilic statins have previously demonstrated higher cellular permeability and, therefore, greater onco-suppressive capacity compared to hydrophilic statins. However, recent studies suggest that the tumor-suppressive effects of statins depend on various factors, including the molecular and histopathological characteristics of tumor cells. Notably, the administration of statins before cancer diagnosis has been associated with the improvement of therapeutic outcomes (11).

Drug discovery is a massively time-consuming, high-risk, and high-priced process (12, 13). Thus, drug repositioning was proposed to reuse existing drugs for a never-considered therapeutic indication. In a crisis like the COVID-19 pandemic, drug repositioning provided a considerable service by using existing drugs for new purposes. For instance, hydroxychloroquine, remdesivir, and ritonavir have been used in different therapeutic indications as antiviral agents (14-15). Besides, in vitro experiments of clinical studies contributed to successful results in the COVID-19 pandemic (15). As Dulak and Jozkowicz suggested, statin drugs play a remarkable role in preventing cancer progression by targeting the blood vessel formation of solid tumors. Even though statins are largely cholesterol-lowering medicines, they are proven anti-cancer agents (16). Hence, this study investigated these proposed statin structures via computational approaches, i.e., machine learning (ML) classification and molecular dynamics (MD) simulation. Primarily, the models establish a quantitative relationship between the chemical structure features of a series of molecules and their biological activities observed in wet laboratory experiments. Recently, several manuscripts utilizing ML modeling have been published. For example, Rajan et al. attempted to develop an ML-powered method that identifies tumor microenvironment (TME) features as predictors of c-Met overexpression status (17). Cruz-Cortes et al. also utilized ML as a general platform for identifying off-target interactions. The applicability of this approach extends to drug discovery, studying statins as inhibitors of the calcium pump SERCA (18). Torabi et al. employed tree-based models to classify and identify potential inhibitors targeting EGF receptors, integrating feature selection, molecular docking, and experimental validation to enhance predictive accuracy and expedite drug discovery (19). Similarly, Arabi et al. utilized tree-based models to identify inhibitors of VEGF receptors, implementing advanced classification techniques, molecular docking, and validation experiments to improve precision and accelerate the discovery process (20).

## 2. Objectives

This study applies ML to simulate c-Met receptor inhibitors, with a particular assessment of the propensity of statin groups to inhibit. LightGBM was used for its effectiveness in handling big data, quick training rate, and high classification accuracy, surpassing other tree-based models by minimizing overfitting and enhancing prediction performance.

## 3. Methods

### 3.1. Machine Learning Step

#### 3.1.1. Molecular Data Preprocessing

During the literature review of c-Met inhibitors, we identified three approved drugs, namely cabozantinib, crizotinib, and foretinib, which exhibit inhibitory effects on the HGF receptor. These drugs were considered crucial compounds and served as positive control groups in our laboratory tests. To develop our ML model, we obtained all tested molecules from PubChem, including those directly associated with the mentioned drugs as positive control groups. Since most of the proposed inhibitors were evaluated using various concentrations of the positive control group in wet-laboratory experiments, the IC_50_ values were standardized by aligning them with the maximum value reported in multiple articles. Due to the large number of reported values across multiple articles, the detailed data has been moved to the Appendices 1 and 2 in the Supplementary File. The IC_50_ values of other related compounds were scaled using the same ratio (Equation 1).

Equation 1.


$$Normalized\;IC_{50}\;compound=IC_{50}\;compound\times \left(\frac{\max\left({IC}_{50}\;standard\;drug\right)}{IC_{50}\;standard,\;article}\right)$$

Accordingly, normalized IC_50_ compound indicated the adjusted IC_50_ value of the compound after normalization to ensure consistency across different sources. IC_50_ compound refers to the original IC_50_ value of the compound before normalization. Moreover, max (IC_50_ standard drug) refers to the maximum IC_50_ value of the standard drug, as reported in Table 1, which is used as a reference for normalization. IC_50_ standard, article expresses the IC_50_ value of the standard drug reported in each specific article, used to scale the IC_50_ values of all compounds within that study.

**Table 1.** The Number of Inhibitor/Weak Inhibitor/Neutral Molecules Used in the Classification Model

Dataset	Standard Value	Number Compound	Number of Inhibitor Molecules	Number of W/N Molecules
**Foretinib**	0.019	549	306	243
**Crizotinib**	0.0137	306	111	195
**Cabozantinib**	0.037	124	44	80
**Total**		979	461	518

Abbreviation: W/N, weak inhibitors/neutral.

Subsequently, the activity of the molecules was normalized, and they were categorized into two distinct groups based on predefined cutoffs: Inhibitors (IC_50_ ≤ 200 nM) and weak inhibitors/neutral (W/N) compounds (IC_50_ > 200 nM). The resulting processed dataset consisted of 980 unique compounds, as outlined in Table 1. 

In the subsequent steps, two distinct sets of molecular descriptors were generated using open-source packages, namely Mordred and RDKit. These packages were utilized to compute structural descriptors such as MORGAN and MACCS fingerprints and physicochemical descriptors. Mordred is a molecular descriptor calculator that generates 2D and 3D descriptors, while RDKit is an open-source cheminformatics toolkit for molecular processing and fingerprint generation. MORGAN, an ECFP-like circular fingerprinting method in RDKit, is used for similarity-based screening, whereas MACCS employs structural key-based fingerprints to encode predefined substructures for molecular similarity assessment. The total number of calculated descriptors can be found in Table 2, along with references to the respective sources (https://github.com/mordred-descriptor/mordred, https://www.rdkit.org/; version 2020.03.1).

**Table 2.** The Number of Molecular Descriptors

Types	Number
**Physicochemical**	
Mordred	1436
Rdkit	174
**Structural**	
MORGAN	921
MACCS	121

As it is well known, the inclusion of redundant variables in ML models can lead to the problem of overfitting and result in a decrease in overall classifier accuracy. Therefore, selecting an appropriate feature selection method in data science is crucial. Additionally, many variables in a model can significantly increase its complexity. Consequently, implementing a variance threshold is an essential baseline approach for feature selection. By removing features that fail to meet a specified threshold of variance, it is assumed that features with higher variance contain valuable information, thus ensuring the utilization of a more practical feature set for the model.

#### 3.1.2. Tree-based Classification

In this study, we propose using a tree-based classifier algorithm. Tree-based algorithms are a prevalent family of supervised learning algorithms renowned for their versatility in addressing classification and regression tasks. These algorithms leverage the inherent tree structure and its various combinations to tackle specific problem domains effectively; notably, XGBoost, CatBoost, LightGBM, Extra Trees, and Random Forest have emerged (21). The performance evaluation of classification algorithms commonly involves using the Stratified K-Fold validation method. This technique is particularly valuable when there is a need to balance the percentage of each class in both the training and testing datasets. Compared to regular K-Fold validation, the critical advantage of Stratified K-Fold is its ability to ensure that each batch of data used for training and testing maintains an equivalent proportion of observations concerning a specific label. Once we identify the best-performing classification model and acquire reliable datasets, we evaluate the potential of statin compounds as inhibitors of the c-Met receptor. By inputting all the statin compounds into the model, we aim to predict their ability to inhibit the c-Met receptor (22).

### 3.2. Molecular Docking

Molecular docking was performed to demonstrate the molecular interactions and prepare input files for further processing. Three-dimensional structural data of c-Met was obtained from the Protein Data Bank (PDB) as a 4XMO PDB file. Removing water and ligands attached to the PDB file and adding hydrogen atoms were done using PyMol 2.3.3 (PyMOL Molecular Graphics System, Version 2.0, Schrodinger, LLC). The 3D conformers of fluvastatin and pitavastatin ligands were downloaded from the www.pubchem.ncbi.nlm.nih.gov website. Ligands were geometry optimized via Gaussian 03 (23). Output PDB files were hydrogenated with the Chimera 1.14 software to be utilized as Autodock 4.2.6 inputs (24, 25). The residues, including M1160, M1211, D1222, M1229, Y1230, V1092, and Y1159, were selected as flexible residues in the active site of the c-Met protein. The protein’s active site was defined as a grid box with 126 × 126 × 126 points and 0.179 Å spacing. The resulting GPF and DPF files generated 500 dock conformation numbers using the Lamarckian genetic algorithm. The conformation with the lowest binding energy of each ligand was selected as the input file for MD simulation.

### 3.3. Molecular Dynamics Simulation

The most stable conformation of each protein-ligand complex was simulated using GROMACS 5.1.2 for 25 ns by GAFF and AMBER03 force fields for protein and ligand molecules, respectively (26, 27). The system was centered in a cubic box solvated with TIP3P water molecules, and the distance between the solute and the box was set to 1 nm (28). The steepest descent algorithm was applied for energy minimization (29). The maximum gradient was set to 1000 kJ/mol/nm to avoid defective geometry and remove incorrect contacts. Two equilibration phases were considered in NVT and NPT ensembles for 300 ps. The Berendsen thermostat was used for temperature coupling at 300 K (30). Pressure coupling was accomplished by the Parrinello-Rahman barostat at 1 bar pressure (31). Long-range electrostatic interactions were computed by the particle mesh Ewald algorithm (32). The production MD run was performed for 25 ns. The integration time step was two fs, and the coordinate trajectories were recorded every 20 ps. VMD 1.9.2 visualizer was used to assay the trajectory files (33). Finally, structural analysis of secondary structure, radius of gyration, hydrogen bond, root-mean-square fluctuation, root-mean-square deviations, and solvent-accessible surface area was performed.

### 3.4. Chemicals and Reagents

Fluvastatin and pitavastatin were obtained from Sigma-Aldrich (St. Louis, MO, USA). Dimethyl sulfoxide (DMSO) was from Merck (Darmstadt, Germany). RPMI 1640, penicillin-streptomycin, and trypsin EDTA were provided by Biosera (East Sussex, UK). Fetal bovine serum (FBS) was bought from Biowest (Nuaille, France). MTT [3-(4,5-dimethylthiazol-2-yl)-2,5-diphenyl tetrazolium bromide] was obtained from Carl-Roth (Germany). Antibodies against Met, p-Met, ERK, and p-ERK were purchased from Cell Signaling Technology, Inc. (Danvers, MA, USA). Akt and p-Akt antibodies were from BioLegend (USA).

### 3.5. Cell Cytotoxicity Evaluation

Human gastric cancer cell lines, AGS and MKN-45, which exhibit c-Met expression, were purchased from the Pasteur Institute (Tehran, Iran) and IBRC Cell Bank (Tehran, Iran), respectively. AGS cells were maintained in RPMI medium supplemented with 10% heat-inactivated FBS and 1% penicillin-streptomycin at 37℃ under a 5% CO_2_ atmosphere. Regarding MKN-45, cells were cultured in RPMI medium with 20% heat-inactivated FBS and 1% penicillin-streptomycin solution. Cells (6 × 10^4^) were seeded in 96-well plates and incubated overnight. Thereafter, the cells were treated with specific concentrations of fluvastatin (0, 3.125, 6.25, 12.5, 25, 50, 100, 200, and 400 µM) and pitavastatin (0, 1.56, 3.125, 6.25, 12.5, 25, 50, 100, and 200 µM) for 48 h. To ensure the inclusion of non-adherent cells in the case of semi-adherent MKN-45 cells, the 96-well plate was centrifuged before treatment and absorbance measurement. Subsequently, the medium was replaced with a 20 µL MTT solution [5 mg/mL in phosphate-buffered saline (PBS)]. Three hours after incubation, formazan crystals were dissolved in 100 µL DMSO, and then optical density at 570/690 nm was measured by an ELISA reader (Anthos, UK) (34). The cell viability was calculated using the following Equation 2 (35).

Equation 2.


$$\%\;Cell\;viabillity=\frac{Mean\;OD\;sample}{Mean\;OD\;blank}\times 100$$

### 3.6. Cell Cycle Analysis

AGS and MKN-45 cells were seeded in 6-well plates and treated with fluvastatin (200 and 400 µM) and pitavastatin (100 and 200 µM) for 48 h. After trypsinization, cells were washed with cold PBS. Fixation of the cells was performed by suspending cells in cold ethanol (70%) at 4ºC for two hours. After centrifugation, 500 µL of propidium iodide (PI) solution (36) was added to the sample pellet and incubated in a dark environment at room temperature for 30 minutes. The FACSCalibur flow cytometer (BD Biosciences, San Jose, CA, USA) quantified the DNA content, and the result was analyzed with FlowJo 7.6.1 software (Tree Star, Inc., Ashland, OR, USA).

### 3.7. Cell Apoptosis Assay

AGS and MKN-45 cells were treated with fluvastatin (200 and 400 µM) and pitavastatin (100 and 200 µM) for 48 h to detect the degree of induced apoptosis. After the incubation time, cells were harvested, washed twice with pre-cooled PBS, and stained with 5 µL of Annexin V-fluorescein isothiocyanate (FITC; Invitrogen, USA) and PI (2 µg/mL; Sigma) for 15 min at room temperature in the dark. Subsequently, the apoptosis of the cells was examined using a FACSCalibur flow cytometer (34).

### 3.8. Western Blot Analysis

MKN-45 (12 × 10^4^/well) and AGS cells (15 × 10^4^/well) were seeded and incubated for 72 h. Then, cells were treated with increasing concentrations of fluvastatin and pitavastatin for 16 h. After exposure, the cells were lysed with lysis buffer (37), resolved on 10% SDS-PAGE, and transferred to the PVDF membrane (Roche, Mannheim, Germany) using a wet transfer system. In the next step, membranes were blocked with 5% skim milk (%w/v) in TBST (1X) buffer for 1.5 h on a shaker at room temperature. Primary antibodies were prepared in 5% bovine serum albumin (BSA), added to the membrane, and incubated overnight at 4ºC on a shaker. After washing the membrane with TBST, membranes were incubated with horseradish peroxidase‐conjugated secondary antibody (Roche) for one hour. Finally, the membranes were developed with a chemiluminescence detection system. The quantitative evaluation of the protein bands was accomplished via ImageJ software and normalized to the corresponding total protein band intensity.

### 3.9. Statistical Analysis

The data obtained were represented as the mean ± standard deviation (SD). Tukey post-hoc test was applied to compare various treatment groups using GraphPad Prism version 5.01 (San Diego, CA). P-values < 0.05 were defined as statistically significant values.

## 4. Results and Discussion

### 4.1. Machine Learning Step

#### 4.1.1. Model Evaluation

The calculated molecular descriptors and their corresponding activity statuses were divided into ten batches to determine the optimal classification model for evaluating statin compounds. Each iteration involved using one batch as the test data, while the remaining batches were utilized for training and validating the model using the Stratified K-Fold technique. This approach ensured a comprehensive assessment of the model's performance. Two statistical methods were employed to ensure reliable comparisons: The *t*-test and Akaike's Information Criterion. The *t*-test assumes that the standard deviations of the two populations being compared are identical. On the other hand, Akaike’s information criterion involves comparing the likelihoods of variable models to detect a better fit to the empirical data.

#### 4.1.2. Investigating Proposed Classification Performance

**Figure 1.**
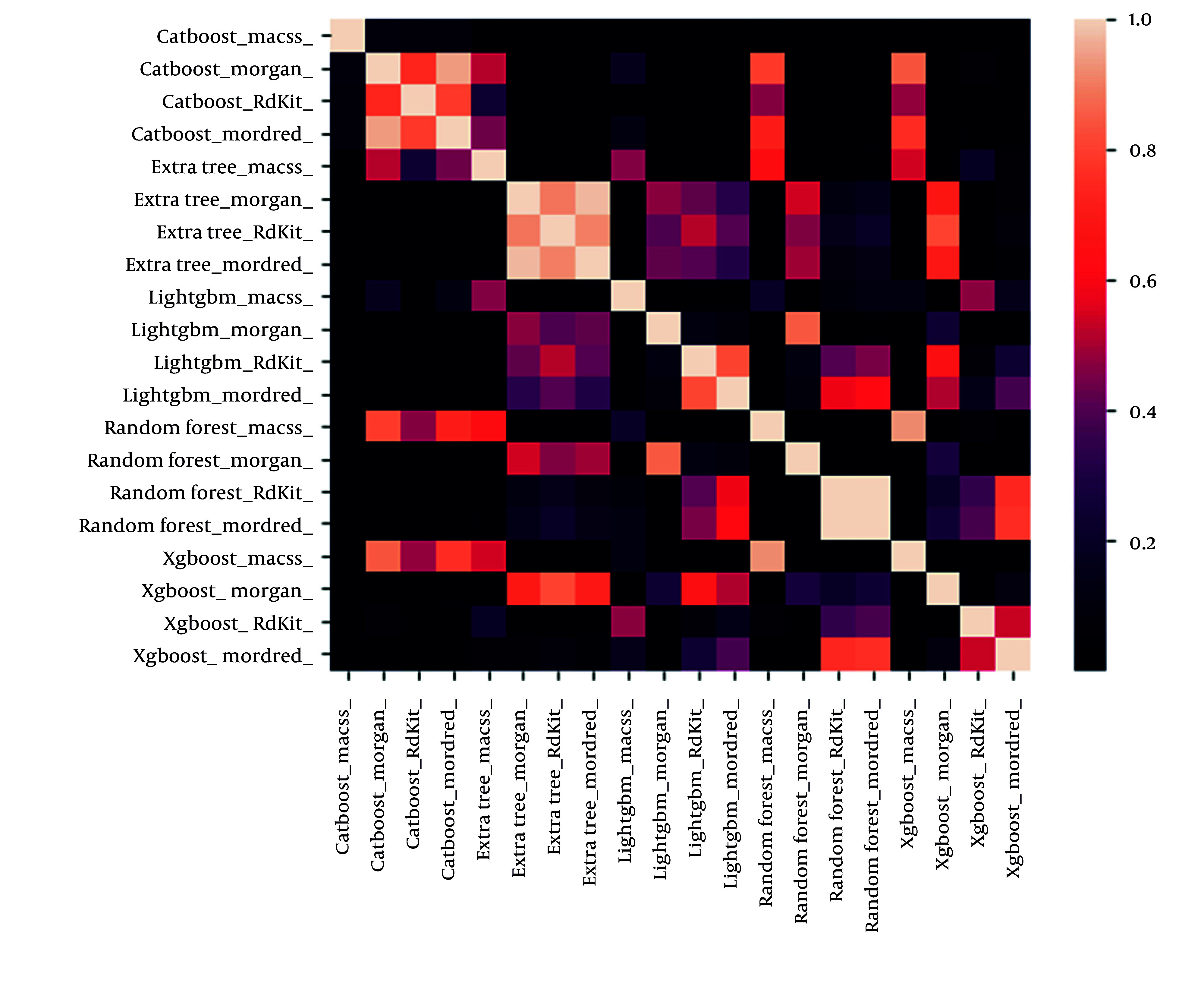
Statistical analysis of the results via the *t*-test method

The *t*-test method (Figure 1) was utilized to perform statistical analysis on the results, with the color indicating the significance value. Based on the *t*-test results, the CatBoost classification algorithm exhibited significantly lower performance than similar algorithms.

**Figure 2.**
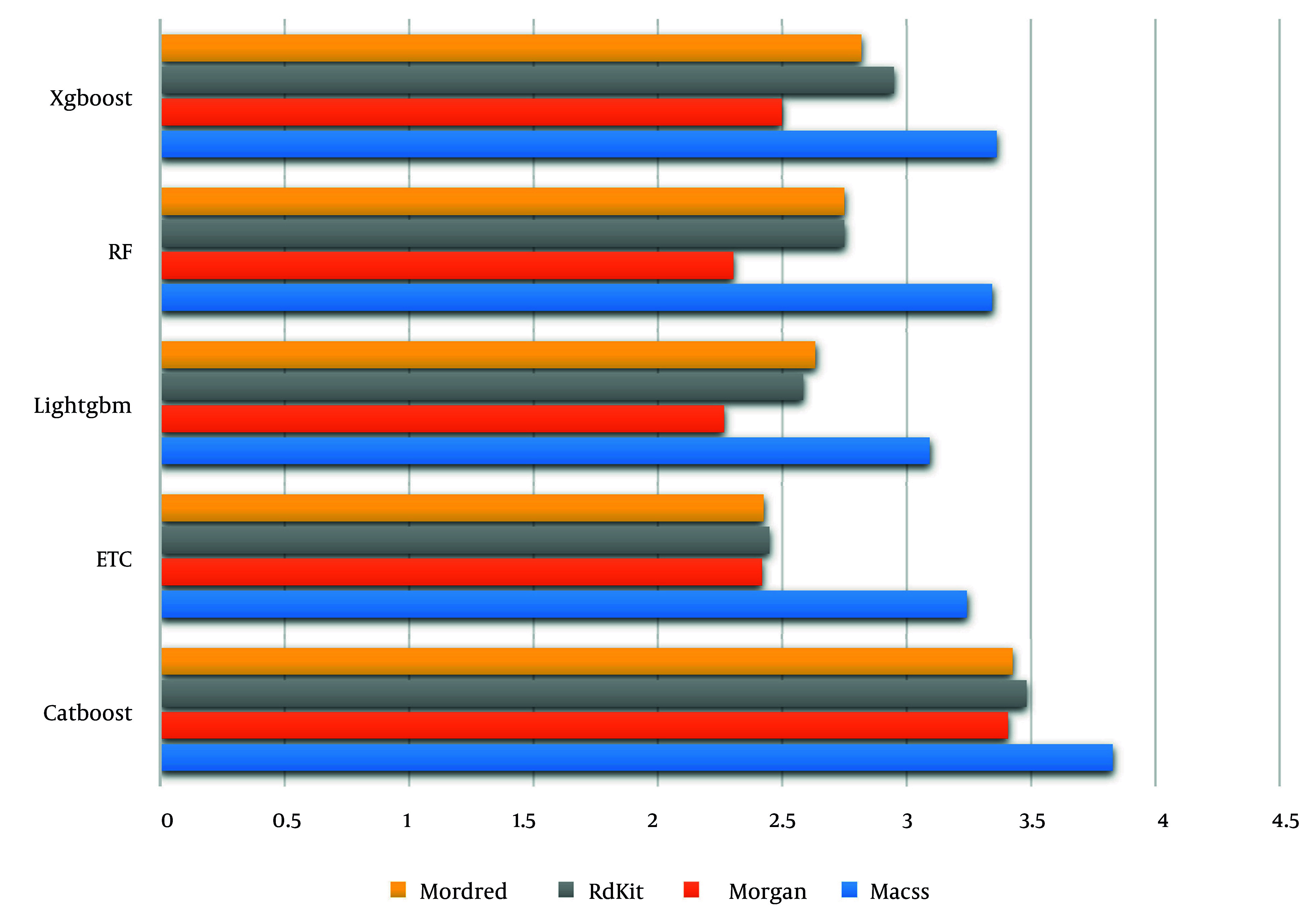
Akaike coefficient values for different tree-based models according to the different molecular fingerprint categories as an input machine learning (ML) model

Besides, the absolute Akaike coefficient was considered to investigate model performance precisely (Figure 2). As displayed, the LightGBM algorithm demonstrated superior performance compared to other models.

**Figure 3.**
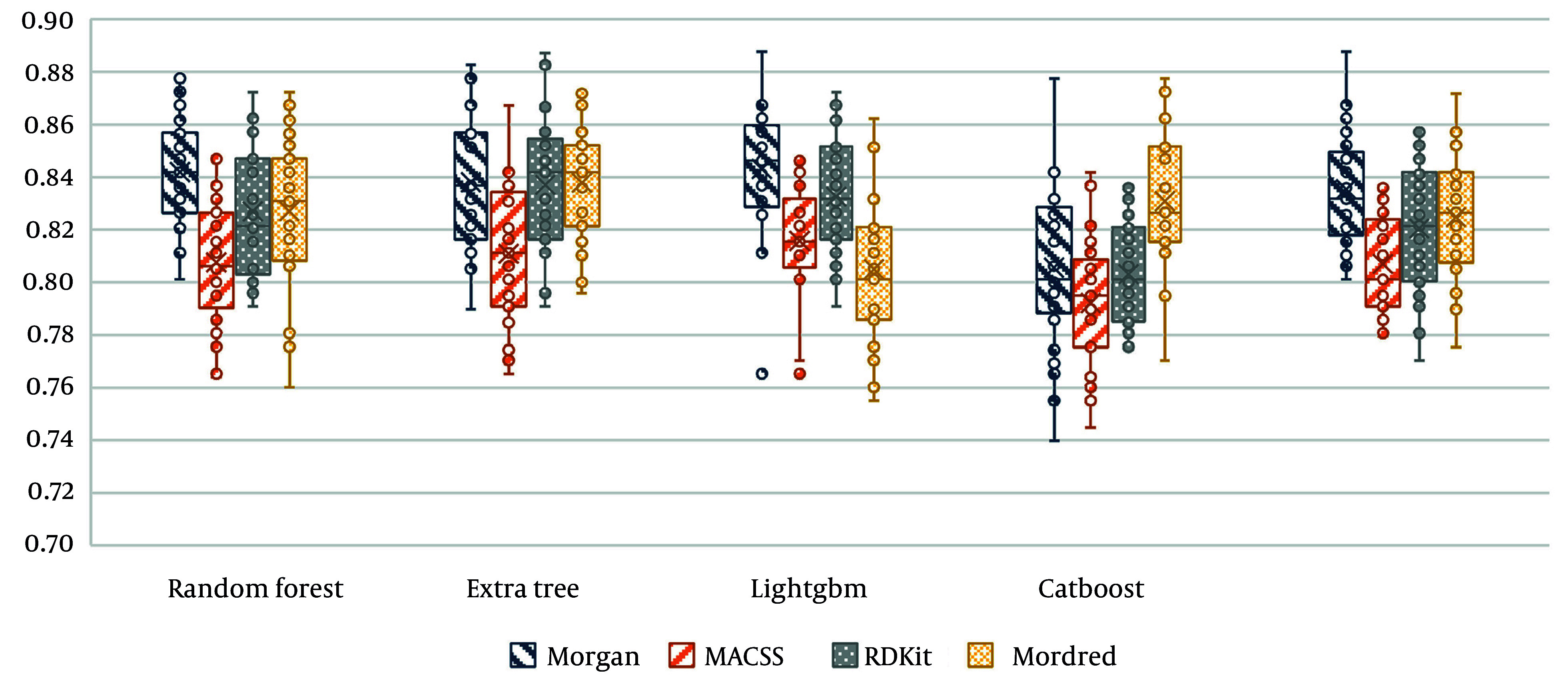
Accuracy values of all proposed classifications based on the different molecular fingerprints as an input model

In the final step, Figure 3 presents the accuracy of all proposed tree-based algorithms based on the four different molecular fingerprints. As displayed, LightGBM performs the best compared to other methods.

CatBoost and LightGBM are both gradient boosting models, but they differ in how they handle categorical variables and training efficiency. CatBoost focuses on encoding categorical features to prevent overfitting, while LightGBM, on the other hand, uses a histogram-based approach and leaf-wise growth strategy, making it significantly faster and more efficient in large datasets. LightGBM outperformed CatBoost in this research, owing to its capacity for high-dimensional molecular descriptors, reduced training time, and better generalization, resulting in enhanced classification efficacy.

**Figure 4.**
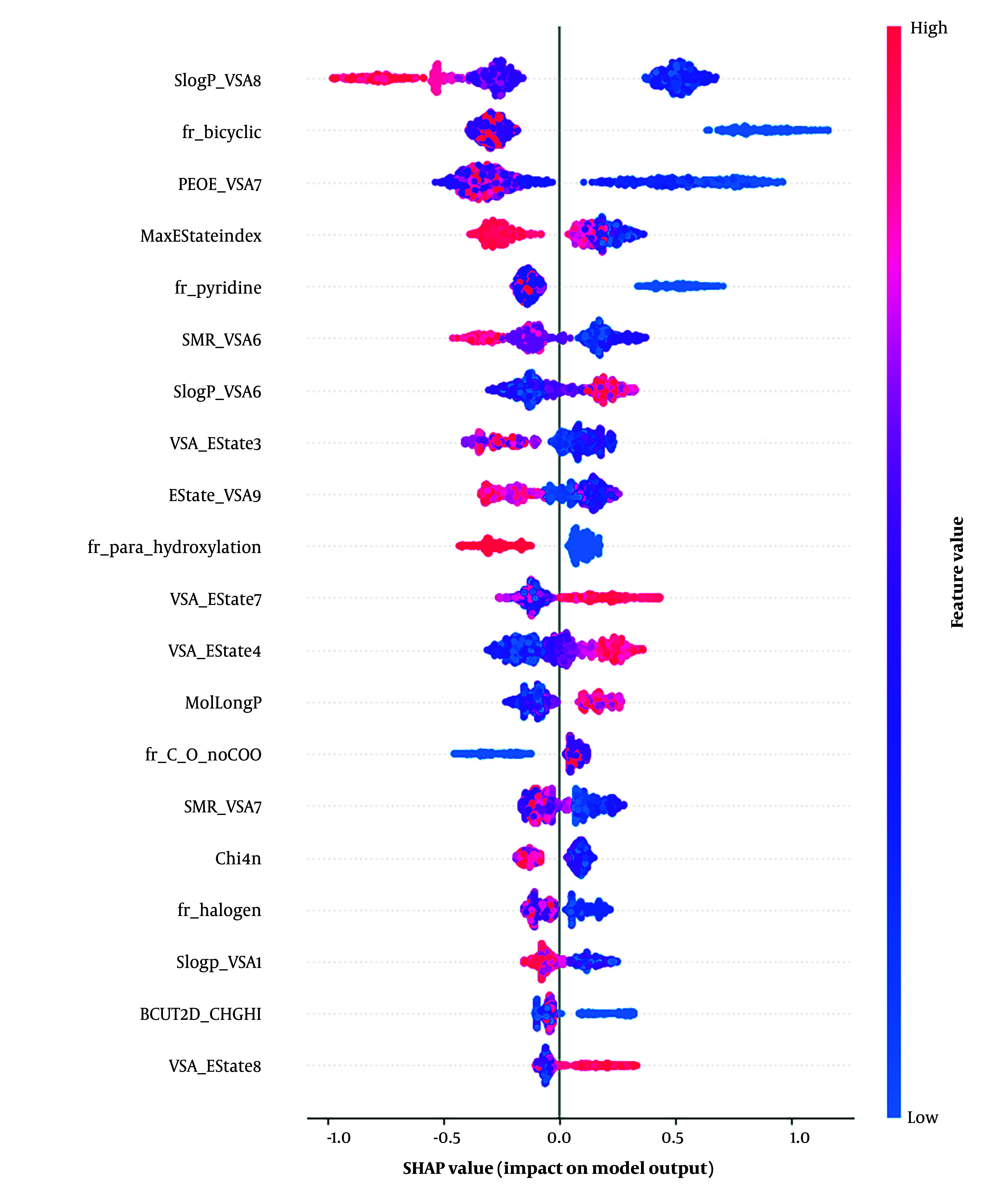
SHAP summary plot highlighting key molecular descriptors influencing LightGBM’s classification of inhibitors and W/N compounds

As shown in Figure 4, to further improve model interpretability, we analyzed feature importance using a SHAP summary plot for the LightGBM model using RDKit descriptors. The top five most influential features were SlogP-VSA8 (lipophilicity), fr-bicyclic (bicyclic structure presence), PEOE-VSA7 (electronic property), maximum E-State Index (max E-State Index), and fr-pyridine (pyridine ring presence). These molecular descriptors were key to distinguishing inhibitors and W/N compounds, showing their importance in active compound discrimination in drug discovery.

Considering both statistical methods, LightGBM emerged as the final algorithm of choice for classifying the physicochemical descriptors of the statin compounds.

#### 4.1.3. Investigating the Proposed Statin Structures by the Proposed Model

Upon completion of the ML phase, we can confidently assert that the model’s performance was reliable in determining the structural viability of statin compounds as potential c-Met inhibitors. The modeling process was repeated 50 times to ensure the reliability of the accuracy of our findings. These repeated iterations enhanced the precision of the model's predictions on statin compounds and mitigated any biases that could impact the results.

During each modeling iteration, we meticulously examined the model’s probability estimation regarding the inhibitory characteristics of a given compound. By assessing the inhibitory probabilities of the compounds across multiple repetitions, we obtained a comprehensive overview of their potential for inhibition. To effectively present these results, we calculated the average inhibition rate for each compound based on its performance in consecutive repetitions. This average inhibition rate is a valuable metric for assessing the compound's overall probability of exhibiting inhibitory activity.

We compiled all relevant information in Table 3 to consolidate our conclusions and provide a clear overview of the predicted inhibition probabilities. This table showcases the compounds’ average inhibition rates, offering valuable insights into their potential as novel c-Met inhibitors. The thorough repetition of the modeling process and subsequent analysis of average inhibition rates have bolstered the reliability of our model's predictions in this context.

**Table 3.** Statin Compound Results

Statins	Number of Weak Inhibitors Predicted	Probability Class Inhibitor	Probability Class Weak Inhibitor
**Pravastatin**	50	0.2878	0.7122
**Pitavastatin**	50	0.3068	0.6932
**Fluvastatin**	50	0.3378	0.6622
**Lovastatin**	50	0.2674	0.7326
**Cerivastatin**	50	0.2698	0.7302
**Simvastatin**	50	0.2612	0.7388
**Atorvastatin**	50	0.2612	0.7388

### 4.2. Molecular Docking

Based on the ML results, two statin compounds, pitavastatin and fluvastatin, were selected for the molecular docking study. Due to the hydrophilicity of pravastatin and research around the anti-cancer property of pravastatin, fractional growth-inhibitory of this ligand has been reported, which led us to choose fluvastatin over pravastatin (38). The most stable position of each molecular docking was selected as an input file for MD simulation. The aforementioned binding energies are -5.3 and -4.9 kJ/mol for pitavastatin and fluvastatin, respectively.

#### 4.2.1. Molecular Dynamics Simulation

**Figure 5.**
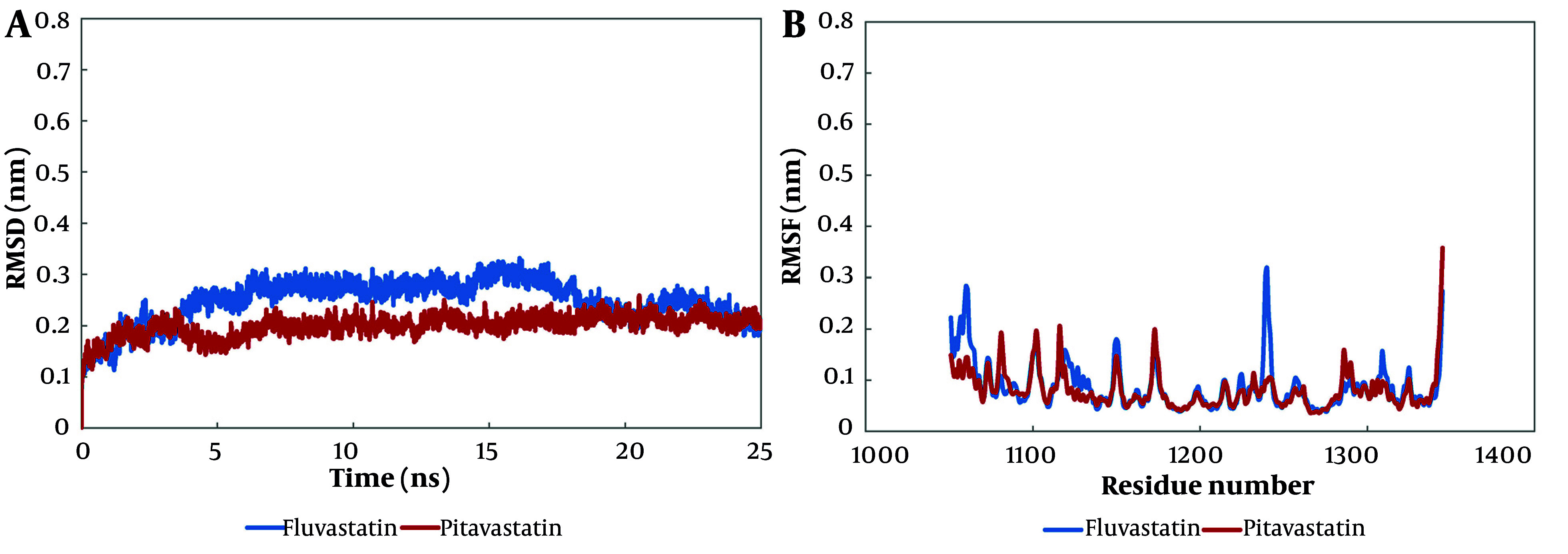
The root mean square deviation (RMSD) and the root mean square fluctuation (RMSF) analyses of molecular dynamics (MD) simulation; both fluvastatin and pitavastatin formed stable complexes at the cellular mesenchymal-epithelial transition (c-Met) ATP-binding site at about 20 ns of simulation: A, the RMSD, and B, RMSF results of alpha carbon atoms of pitavastatin-c-Met complex and fluvastatin-c-Met complex.

The MD simulation was utilized to exhibit the molecular interactions at the protein-ligand complexes and evaluate the binding mode (Appendix 1 in Supplementary File). The backbone root mean square deviation (RMSD) analysis investigates the stability of a system and its conformational behavior. The RMSD value of fluvastatin leveled off at 0.2 nm from nearly 20 ns until the end of the simulation, whereas the RMSD value of pitavastatin stood at about 0.2 nm from 8 ns to 25 ns of simulation (Figure 5A). 

The RMSF analysis represents the average fluctuation of protein residues compared to its RMSF over MD simulation time. The RMSF value of the fluvastatin complex showed that residues 5 to 15, 185 to 195, and 295 to 300 had the highest fluctuations (0.3 ± 0.2 nm), while the majority of residues fluctuated at about 0.6 nm. In addition, the preponderance of pitavastatin complex residues fluctuated at about 0.6 nm, while residues from 295 to 300 had the highest fluctuations at 0.34 nm (Figure 5B). 

The radius of gyration is a rough measure of the compactness of a protein structure. A low radius represents a stable and folded system. According to the data, both complexes exhibited slight compactness, which illustrates the stabilization of the system (detailed data is not displayed here, but the average Rg value is available in Table 4). 

**Table 4.** Comparison of Hydrogen Bond Occupancy Percentages ≥ 30 and Gyration Radius

Ligand; Intermolecular H-Bond	H-Bond Occupancy (%)	Rg (nm)
**Fluvastatin**		2.00
K 1110: NZ::O_2_	32.68	
Y 1230: OH::O	99.44	
D 1222: O_1_::OD_2_	36.17	
**Pitavastatin**		
R 1086: NE::O_2_	30.03	2.01

#### 4.2.2. Hydrogen Bond

Hydrogen bond analysis determines the number of hydrogen bonds and their duration in the simulated systems. According to Table 4, intermolecular H-bonds between fluvastatin and the c-Met protein are greater in number and duration compared to pitavastatin. Based on this analysis, both complexes are qualified to maintain a stable system. The hydrogen bond occupancy percentage determines the certainty of hydrogen bond formation between the ligand and the receptor during the simulation time. According to the average of hydrogen bond occupancies in fluvastatin and pitavastatin complexes, hydrogen bonds with ≥ 30% occupancy were evaluated (39, 40). Furthermore, fluvastatin has formed more H-bonds compared to pitavastatin. Based on hydrogen bond occupancy and hydrophobic interaction analysis, pitavastatin makes hydrogen bonds with R1086 and V1083 residues.

According to Peach et al.’s study on agents targeting the c-Met tyrosine kinase domain, there are four main interactions at the ATP-binding site of the c-Met protein: (1) Hydrogen bond with hinge region residues (M1160 and P1158); (2) aromatic or hydrophobic interaction in the hydrophobic pocket; (3) hydrophobic interaction in the hydrophobic sub-pockets; (4) hydrogen bond with Y1230 backbone in the activation loop or π-stacking interaction of an aromatic group with the Y1230 ring.

**Figure 6.**
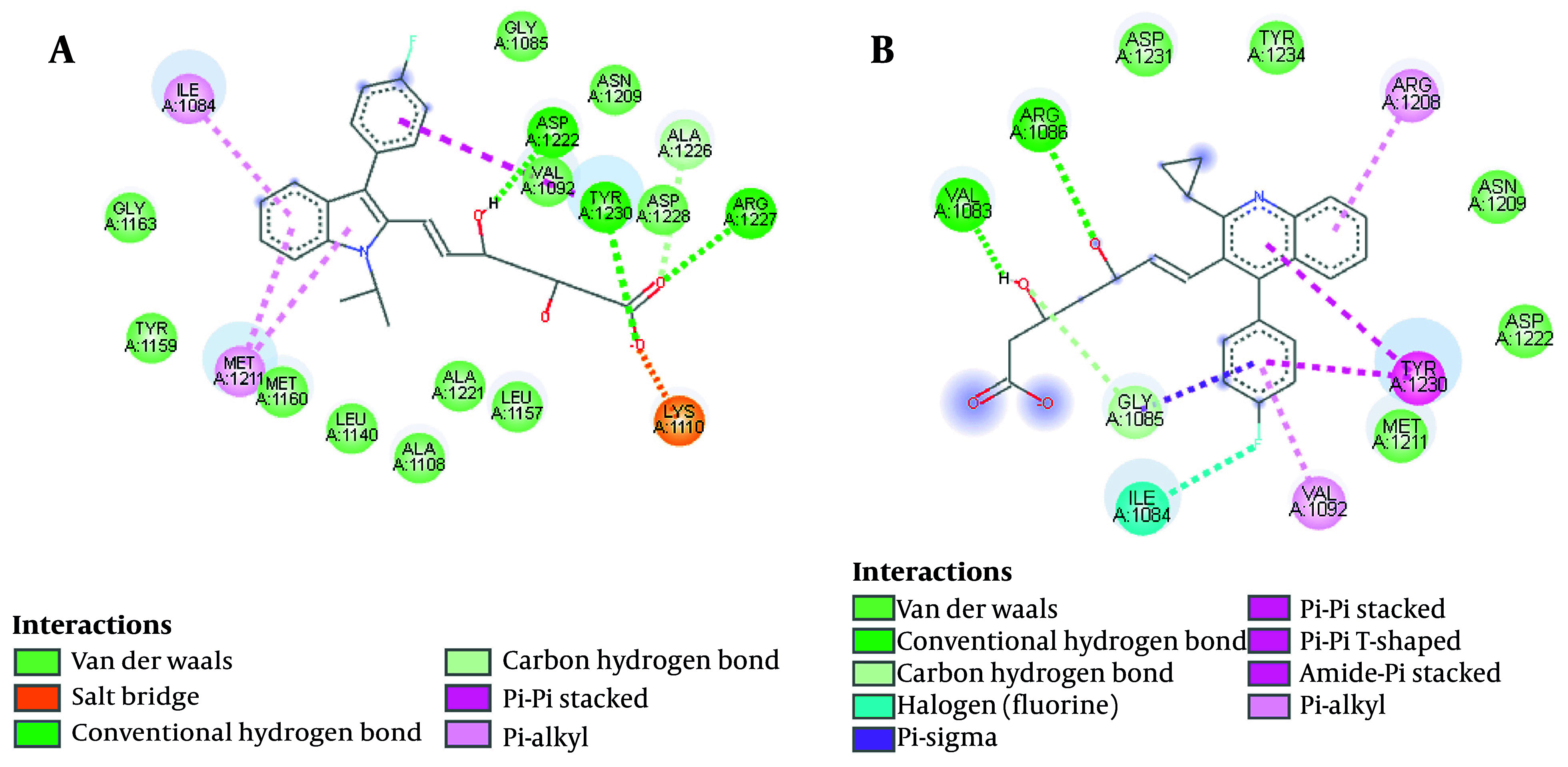
Hydrogen and hydrophobic interactions between A, fluvastatin and B, pitavastatin at the cellular mesenchymal-epithelial transition (c-Met) receptor ATP-binding site

Almost all ATP-competitive small molecule inhibitors for the kinase domain form hydrogen bonds with P1158 and M1160 at the hinge region (where the N-terminal of the β region meets the C-terminal, forming a hydrophobic pocket) (41). Peach et al. also declare that the high binding affinity of a compound to the c-Met ATP-binding site is due to hydrogen bond formation with the Y1230 residue (Appendix 2 in Supplementary File). Triazolotriazine has shown activity as a c-Met inhibitor. An aryl group attached to the triazine ring and an acceptable hydrogen bond acceptor linked to the pendant benzyl ring are necessary features of the c-Met inhibitor. However, phenol acts as a hinge binder (with M1160), and the triazine interacts with Y1230. Salt bridge interaction is an eminent force notable for its strength and stability. Using the BIOVIA Discovery Studio Visualizer (42), the salt bridge formation is depicted (Figure 6). Accordingly, fluvastatin possessed a salt bridge with the c-Met receptor (A1086), whereas neither pi-sulfur nor salt bridge is reported in pitavastatin. Besides, a π-π interaction is reported between pitavastatin and the Y1230 residue (activation loop).

#### 4.2.3. Hydrophobic Interactions

Hydrophobic interactions and hydrogen bonds are proposed to determine the folding of proteins and their stability in complexes with ligands. The BIOVIA Discovery Studio Visualizer (42) was utilized to represent meaningful electrostatic and non-electrostatic interactions of pitavastatin and fluvastatin with the c-Met receptor ATP-binding site from 20 ns to 25 ns of stimulation. As illustrated in Figure 6, the ligand makes hydrogen bonds with D1222, Y1230, and R1227 in the fluvastatin-c-Met complex. Furthermore, interactions with I1084, K1110 (hydrophobic sub-pocket), M1211 (central hydrophobic pocket), and Y1230 residues (activation loop) were reported. Pitavastatin makes hydrogen bonds with V1083 and A1086 residues. Additionally, the presence of fluorine halogen was reported. Figure 6 depicts N1209, M1211 (central hydrophobic pocket), D1222, D1231, and Y1234 residues forming hydrophobic interactions with pitavastatin. Moreover, a π-π interaction was seen between Y1230 (activation loop) and pitavastatin.

As Damghani et al. have reported, c-Met possesses an ATP-binding site and an allosteric site that is contiguous to the ATP site. Type I inhibitors occupy the ATP site, while type II inhibitors occupy both the ATP and allosteric sites. The amino acid residues around the bound type I ligands are generally D1164, I1084, G1085, H1162, G1163, K1161, Y1159, M1160, A1108, P1158, M1211, K1110, V1092, L1157, A1221, L1140, A1226, D1222, N1209, R1208, and Y1230. Therefore, our ligands’ hydrophobic interactions, hydrogen bonds, and π-π interactions can be accounted for (43).

### 4.3. In Vitro Results

#### 4.3.1. Cytotoxicity Effect of Fluvastatin and Pitavastatin on Gastric Cancer Cells

**Figure 7.**
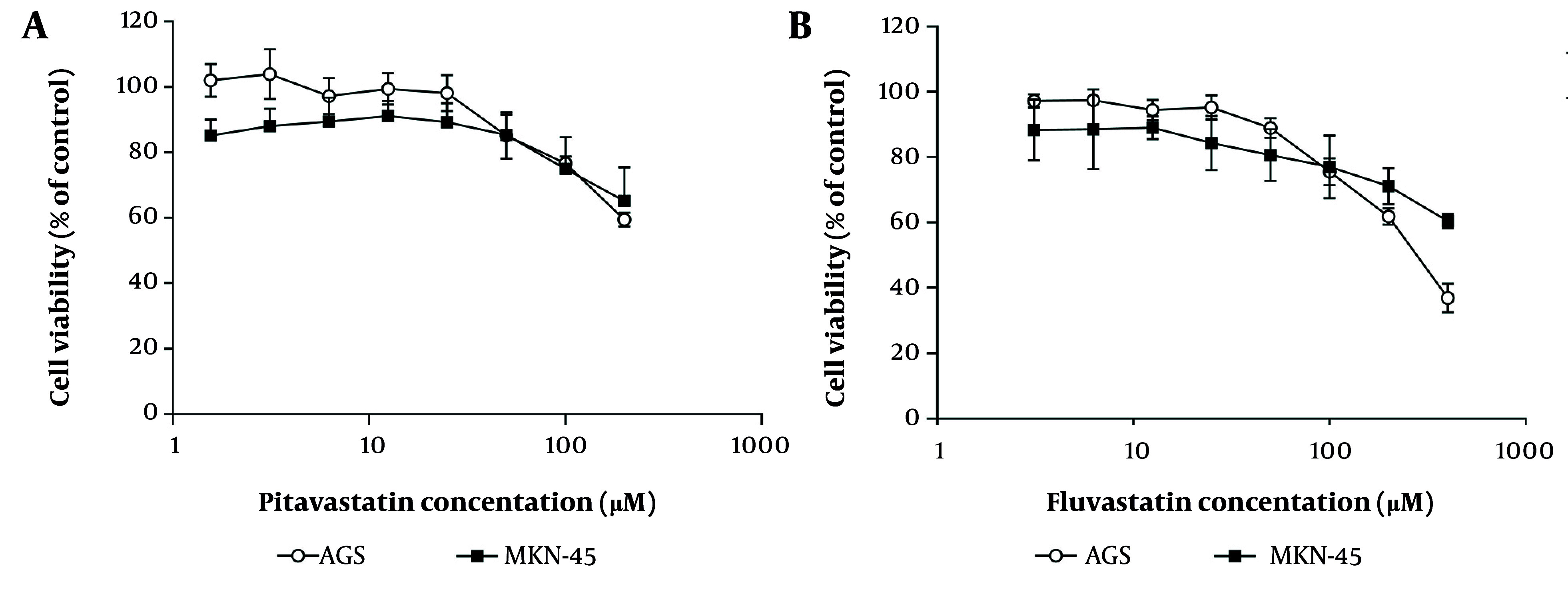
The half-maximal inhibitory concentration of pitavastatin and fluvastatin on AGS and MKN-45 cell lines after 48 hours of treatment

The MTT assay determined that fluvastatin abolished AGS cell growth with an IC_50_ value of 230 ± 25 µM after 48 h of treatment; however, this inhibitory activity could not be observed in the case of MKN-45 cells at 400 µM (Figure 7). After administering variable concentrations of pitavastatin on both cell lines for 48 h, a maximum cytotoxicity effect of 40% was obtained at 200 µM. Although no apparent toxic effects (less than IC_50_) were noted in AGS and MKN-45 cells following pitavastatin treatment, Wang et al. have reported the cytotoxic effects of pitavastatin on cancer cell lines. They revealed that following treatment of 4T1.2 and 4T1 mouse mammary tumor cells and MDA-MB-231 human breast cancer cells with pitavastatin, proliferation and migration of tumor cells were inhibited through down-regulation of signaling pathways mediated via mevalonate and PPAR-γ. Consequently, inhibiting Snail and MMP-9 leads to the reversal of epithelial-mesenchymal transition (EMT) (44).

#### 4.3.2. Effect of Fluvastatin and Pitavastatin on Cell Cycle Distribution

**Figure 8.**
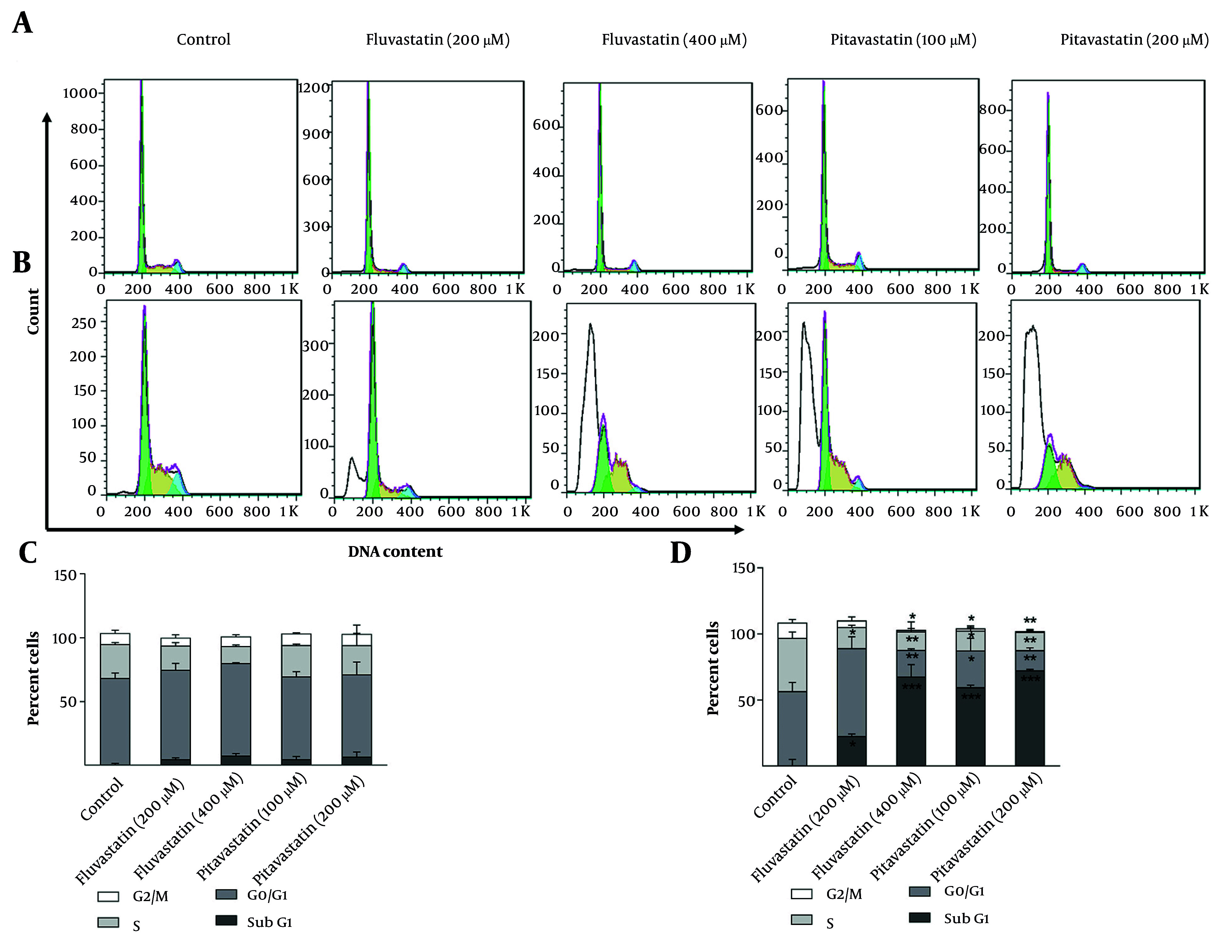
The percentage of cells in different cell cycle phases: Two-dimensional graph of cell cycle analysis resulted from flow cytometry for A and C, MKN-45 and B and D, AGS cells, treated with fluvastatin and pitavastatin concentrations after 48 hours in comparison with untreated cells (control group; * P < 0.05, ** P < 0.01, and *** P < 0.001 relative to controls).

MKN-45 and AGS cells were treated with fluvastatin (200 and 400 µM) and pitavastatin (100 and 200 µM) for 48 h. Cell cycle arrest could not be observed during the process in MKN-45 cells. According to Figure 8A-C, a slight increase in the sub-G1 phase and a trivial decrease in the S phase are observed in the MKN-45 cell line treated with fluvastatin and pitavastatin. Alternatively, a perceptible decrease in S phase progression is observed due to concentration increases in AGS cell lines. Cells at the sub-G1 phase were reported following the concentration increase in both cell lines, which registers cell apoptosis. AGS cells treated with fluvastatin at 200 µM demonstrated a slight increase (P < 0.05) in G0/G1 phase cells (60.5%) in comparison with the control group (55.18%; Figures 8B-D). The increase in the sub-G1 phase is associated with enhanced apoptosis in AGS cells treated with fluvastatin 200 µM (45). Fluvastatin 400 µM administration resulted in a significant decrease in G1, S, and G2 phase percentages in AGS cells. Notably, 61.49% of cells confront apoptotic cell death in comparison to the control group (2.68%), which is statistically significant (P < 0.01). It has also been revealed that AGS treatment by pitavastatin 100 µM displayed a remarkable decrease in the G1, S, and G2 phases. Comparison of the sub-G1 frequency value of pitavastatin 100 µM (60.86%) with the control group (2.68%) highlights the increased apoptosis in AGS cells. However, pitavastatin 200 µM exhibited higher apoptotic cell death (71.79%) than pitavastatin 100 µM. In another study, Nagayama et al. revealed that pitavastatin treatment leads to cell cycle arrest and apoptosis due to the cell cycle regulator p21 upregulation and NF-κB inhibition in different cancer cells (46).

These data suggest that fluvastatin 400 µM and pitavastatin 200 µM suppressed the AGS cell cycle progression remarkably and increased cell apoptosis explicitly. In contrast, both tested drugs displayed trivial changes in the cell cycle distribution of MKN-45 cells (Figures 8A-C).

#### 4.3.3. Apoptotic Effect of Fluvastatin and Pitavastatin on Gastric Cancer Cells

**Figure 9.**
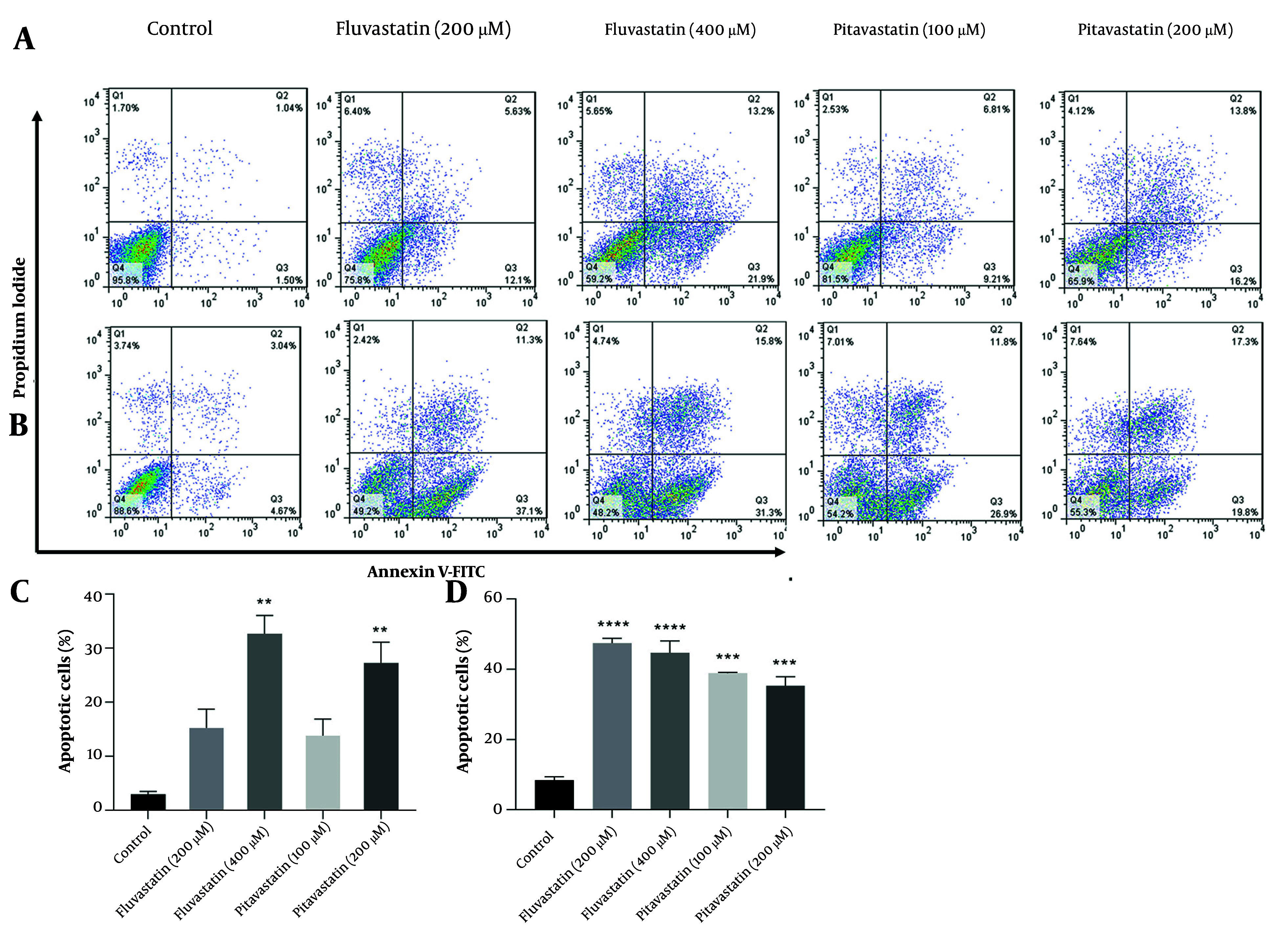
The percentage of apoptotic cells for A and C, MKN-45 cells and B and D, AGS cells was treated with various concentrations of fluvastatin and pitavastatin for 48 hours using Annexin V-fluorescein isothiocyanate (FITC)/propidium iodide (PI) double staining [** P < 0.01, *** P < 0.001, and **** P < 0.0001 as compared to untreated cells (control group)].

Figure 9A-D depicts fluvastatin-induced total apoptosis at 400 µM concentrations (32.70%, P < 0.01) on the MKN-45 cells. This programmed cell death aligns with previous reports of breast cancer (47). However, Jiang et al. found that fluvastatin caused no apoptosis induction towards gastric cancerous cells (48). MKN-45 cells underwent 27.3% total apoptosis at 200 µM of pitavastatin (P < 0.01). In the case of AGS cells, total apoptosis at pitavastatin 100 and 200 µM is about 38.85% and 35.25% (P < 0.001), respectively. A significant increase in the number of cells undergoing apoptosis can be registered at fluvastatin 200 and 400 µM (47.45% and 44.70%, respectively; P < 0.0001) compared to the control group. Previous studies have revealed that pitavastatin activates caspases in vitro and inhibits tumor growth in xenograft models in OSCC and ESCC, breast, glioblastoma, liver, colon, ovarian, and pancreatic cancers (46). Sahebkar et al. confirmed that pitavastatin also increased cancer cell apoptosis by increasing the cleavage of apoptosis-related proteins, including caspase-9 and caspase-3 (49).

#### 4.3.4. Western Blot

The c-Met activation occurs through its constitutive phosphorylation, which results in the modification of several signaling pathways. These signaling pathways are engaged in cell proliferation, growth, and survival (50-52). This study explored whether fluvastatin and pitavastatin inhibit c-Met phosphorylation in MKN-45 (c-Met amplified) and AGS cell lines.

**Figure 10.**
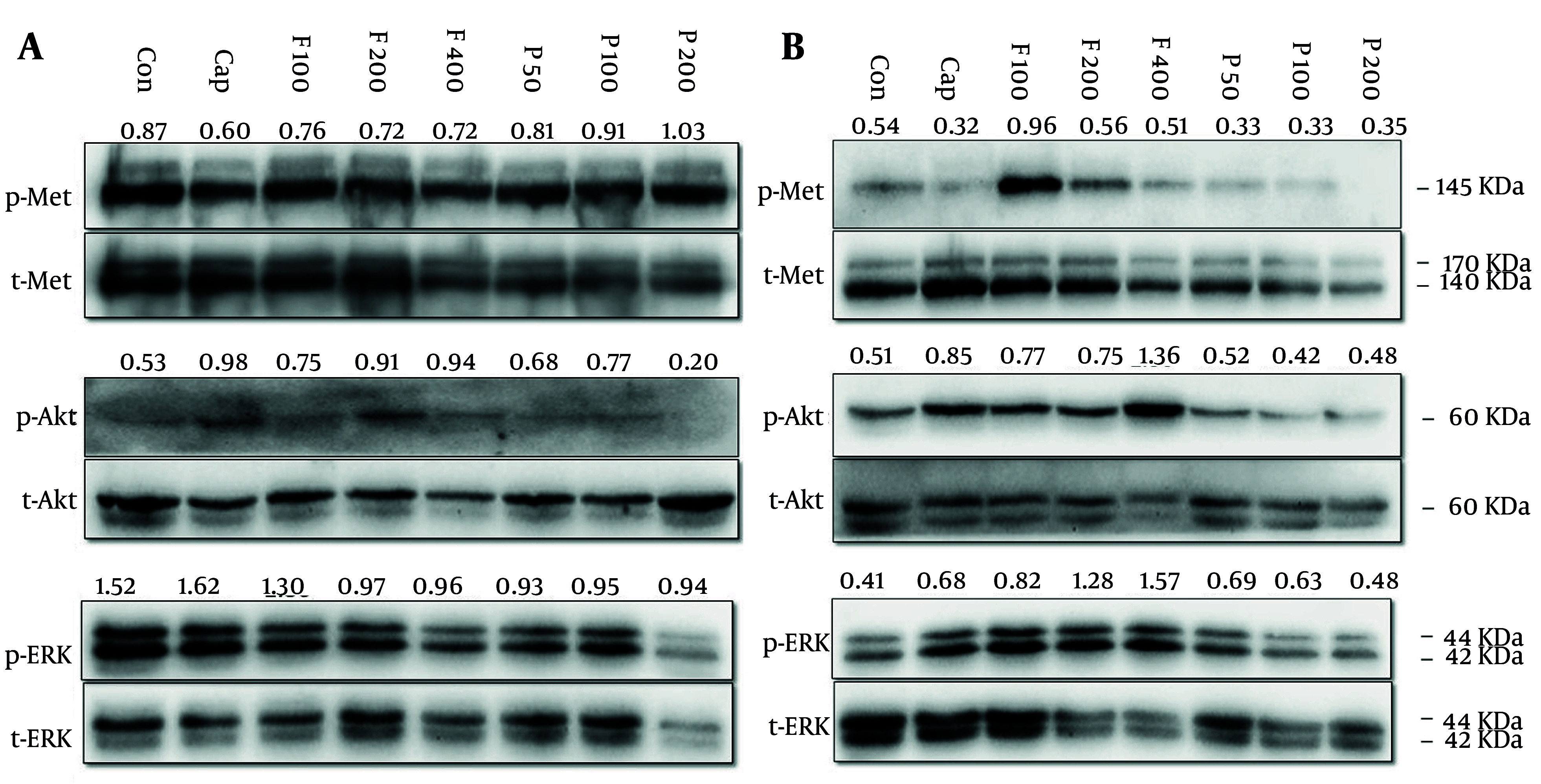
A, MKN-45 and B, AGS cells were treated with increasing concentrations of fluvastatin (F; 100, 200, and 400 µM), pitavastatin (P; 50, 100, and 200 µM), and capmatinb (Cap; 2 nM) for 48 hours. Subsequently, cells were lysed and resolved on SDS-PAGE, and target proteins were detected by the western blotting technique, as mentioned in the method section.

MKN-45 and AGS cell lines were treated with pitavastatin (50, 100, and 200 μM) and fluvastatin (100, 200, and 400 μM). As illustrated in Figure 10, capmatinib, a selective c-Met inhibitor (Cayman Chemical; USA), decreased c-Met phosphorylation, and fluvastatin exhibited a negligible decrease in Met phosphorylation in MKN-45 cells. Also, a slight p-Met increase can be observed in cells treated with pitavastatin in a dose-dependent manner (Figure 10A). In AGS cells, c-Met phosphorylation was only dramatically enhanced in 100 μM fluvastatin but not in other doses. However, pitavastatin decreased its phosphorylation compared with the control group (Figure 10B). The c-Met that has undergone processing forms dimers on the cell membrane and binds to HGF via its sema domain. Subsequently, the stimulation of Met by HGF triggers the activation of the PI3K/Akt, RAS, and ERK signaling pathways, which facilitate cell growth and migration (53).

Fluvastatin and pitavastatin enhanced Akt and reduced ERK phosphorylation in MKN-45 cells, respectively. Fluvastatin also increased Akt phosphorylation, especially at the highest dose (400 μM), but in pitavastatin-treated cells, we cannot see a significant change relative to the control group. Our data displayed that fluvastatin, but not pitavastatin, increased ERK phosphorylation dose-dependently.

Accordingly, Xu et al. discovered that pitavastatin suppressed tumor development by reducing the activity of Akt and ERK signaling pathways via the disruption of immature Met induced by malfunctioning of the Golgi apparatus. Additionally, they found that the expression of GGPS1 played a crucial role in determining the susceptibility to cell growth suppression by pitavastatin and other statins. Furthermore, their research demonstrated that the combination of pitavastatin and capmatinib resulted in a more pronounced reduction of oral and esophageal tumor development than pitavastatin alone due to complete Met signaling inhibition (54).

### 4.4. Conclusions

The present study sought to investigate the c-Met effect on poor-prognosis cancers by statin therapy. Statins affect cell proliferation, survival, and mobility via different mechanisms of action. The findings of the ML step illustrated that the statin family structures had a low probability of inhibiting the c-Met receptor due to their inactivity at the binding site. Since the ML step, accompanied by the MD method, provides more subtle results in recent searches, fluvastatin and pitavastatin with higher inhibiting probability (probability class inhibitor > 0.3) were selected for the MD process and in vitro analysis to evaluate the ML data theoretically and experimentally. Both fluvastatin and pitavastatin exhibited cytotoxicity, apoptosis induction, and sub-G1 phase accumulation in AGS and MKN-45 cells. The protein expression analysis indicated that the tested statin compounds have limited potential as c-Met inhibitors, consistent with ML results. While our findings did not confirm that statins directly inhibit the c-Met pathway, they uncovered cellular responses and computational predictions suggesting a possible interaction. Further studies on statin treatment in cancerous cells are needed to elucidate the precise mechanism of inhibition.

ijpr-24-1-158845-s001.pdf

## Data Availability

The dataset presented in this study are uploaded during submission as a supplementary file and are openly available for readers upon request.

## References

[1] Anitha K, Dua K, Chellappan DK, Gupta G, Singh SK, Lakshmi SM (2023). HGF/c-MET: A Potential Target for the Treatment of Various Cancers.. Curr Enzyme Inhib..

[2] Mwale C, Sunaga T, Wang Y, Bwalya EC, Wijekoon HMS, Kim S (2023). In vitro chondrotoxicity of bupivacaine, levobupivacaine and ropivacaine and their effects on caspase activity in cultured canine articular chondrocytes.. J Vet Med Sci..

[3] Pang Y, Lu T, Xu-Monette ZY, Young KH (2023). Metabolic Reprogramming and Potential Therapeutic Targets in Lymphoma.. Int J Mol Sci..

[4] Damghani T, Moosavi F, Khoshneviszadeh M, Mortazavi M, Pirhadi S, Kayani Z (2021). Imidazopyridine hydrazone derivatives exert antiproliferative effect on lung and pancreatic cancer cells and potentially inhibit receptor tyrosine kinases including c-Met.. Sci Rep..

[5] Stryjkowska-Gora A, Karczmarek-Borowska B, Gora T, Krawczak K (2015). Statins and cancers.. Contemp Oncol..

[6] Arnold M, Abnet CC, Neale RE, Vignat J, Giovannucci EL, McGlynn KA (2020). Global Burden of 5 Major Types of Gastrointestinal Cancer.. Gastroenterol..

[7] Wang F, Shen L, Li J, Zhou Z, Liang H, Zhang X (2019). The Chinese Society of Clinical Oncology (CSCO): clinical guidelines for the diagnosis and treatment of gastric cancer.. Cancer Commun..

[8] Fuse N, Kuboki Y, Kuwata T, Nishina T, Kadowaki S, Shinozaki E (2016). Prognostic impact of HER2, EGFR, and c-MET status on overall survival of advanced gastric cancer patients.. Gastric Cancer..

[9] Ji P, Chen T, Li C, Zhang J, Li X, Zhu H (2025). Comprehensive review of signaling pathways and therapeutic targets in gastrointestinal cancers.. Crit Rev Oncol Hematol..

[10] Faulkner R, Jo Y (2022). Synthesis, function, and regulation of sterol and nonsterol isoprenoids.. Front Mol Biosci..

[11] Filaferro L, Zaccarelli F, Niccolini GF, Colizza A, Zoccali F, Grasso M (2024). Are statins onco- suppressive agents for every type of tumor? A systematic review of literature.. Expert Rev Anticancer Ther..

[12] DiMasi JA, Faden LB (2011). Competitiveness in follow-on drug R&D: a race or imitation?. Nat Rev Drug Discov..

[13] Carter PH, Berndt ER, DiMasi JA, Trusheim M (2016). Investigating investment in biopharmaceutical R&D.. Nat Rev Drug Discov..

[14] Rolain JM, Colson P, Raoult D (2007). Recycling of chloroquine and its hydroxyl analogue to face bacterial, fungal and viral infections in the 21st century.. Int J Antimicrob Agents..

[15] Takla M, Jeevaratnam K (2020). Chloroquine, hydroxychloroquine, and COVID-19: Systematic review and narrative synthesis of efficacy and safety.. Saudi Pharm J..

[16] Dulak J, Jozkowicz A (2005). Anti-angiogenic and anti-inflammatory effects of statins: relevance to anti-cancer therapy.. Curr Cancer Drug Targets..

[17] Rajan D, Egger R, Rahsepar B, Fahy D, Wapinski I, Glass B (2022). P2.07-01 Deep-Learning Based Prediction of c-MET Status from Digitized H&E-Stained Non-Small Cell Lung Cancer Tissue Samples.. J Thorac Oncol..

[18] Cruz-Cortes C, Velasco-Saavedra MA, Fernandez-de Gortari E, Guerrero-Serna G, Aguayo-Ortiz R, Espinoza-Fonseca LM (2023). A novel machine learning-based screening identifies statins as inhibitors of the calcium pump SERCA.. J Biol Chem..

[19] Torabi M, Yasami-Khiabani S, Sardari S, Golkar M, Perez-Sanchez H, Ghasemi F (2024). Identification of new potential candidates to inhibit EGF via machine learning algorithm.. Eur J Pharmacol..

[20] Arabi N, Torabi MR, Fassihi A, Ghasemi F (2024). Identification of potential vascular endothelial growth factor receptor inhibitors via tree‐based learning modeling and molecular docking simulation.. J Chemom..

[21] Motamedi F, Perez-Sanchez H, Mehridehnavi A, Fassihi A, Ghasemi F (2022). Accelerating Big Data Analysis through LASSO-Random Forest Algorithm in QSAR Studies.. Bioinformatics..

[22] Ghasemi F, Mehri A, Peña-García J, den-Haan H, Pérez-Garrido A, Fassihi A, Ortuño F, Rojas I (2015). Improving Activity Prediction of Adenosine A2B Receptor Antagonists by Nonlinear Models.. Bioinformatics and Biomedical Engineering..

[23] Frisch MJEA, Trucks GW, Schlegel HB, Scuseria GE, Robb MA, Cheeseman JR (2009). gaussian 09, Gaussian. Inc., Wallingford CT..

[24] Goodsell DS, Morris GM, Olson AJ (1996). Automated docking of flexible ligands: Applications of autodock.. J Mol Recognit..

[25] Pettersen EF, Goddard TD, Huang CC, Couch GS, Greenblatt DM, Meng EC (2004). UCSF Chimera--a visualization system for exploratory research and analysis.. J Comput Chem..

[26] Pronk S, Pall S, Schulz R, Larsson P, Bjelkmar P, Apostolov R (2013). GROMACS 4.5: a high-throughput and highly parallel open source molecular simulation toolkit.. Bioinformatics..

[27] Wang J, Wolf RM, Caldwell JW, Kollman PA, Case DA (2004). Development and testing of a general amber force field.. J Comput Chem..

[28] Jorgensen WL, Chandrasekhar J, Madura JD, Impey RW, Klein ML (1983). Comparison of simple potential functions for simulating liquid water.. J Chem Phys..

[29] Fliege J, Svaiter BF (2000). Steepest descent methods for multicriteria optimization.. Math Oper Res..

[30] Lemak AS, Balabaev NK (1994). On The Berendsen Thermostat.. Mol Simul..

[31] Parrinello M, Rahman A (1981). Polymorphic transitions in single crystals: A new molecular dynamics method.. J Appl Phys..

[32] Essmann U, Perera L, Berkowitz ML, Darden T, Lee H, Pedersen LG (1995). A smooth particle mesh Ewald method.. J Chem Phys..

[33] Humphrey W, Dalke A, Schulten K (1996). VMD: visual molecular dynamics.. J Mol Graph..

[34] Aliebrahimi S, Farnoudian-Habibi A, Heidari F, Amani A, Montazeri V, Sabz Andam S (2024). Using chitosan-coated magnetite nanoparticles as a drug carrier for opioid delivery against breast cancer.. Pharm Dev Technol..

[35] Kamiloglu S, Sari G, Ozdal T, Capanoglu E (2020). Guidelines for cell viability assays.. Food Frontiers..

[36] Majdzadeh M, Aliebrahimi S, Vatankhah M, Ostad SN (2017). Effects of celecoxib and L-NAME on apoptosis and cell cycle ofMCF-7 CD44+/CD24–/low subpopulation.. Turk J Biol..

[37] Gholizadeh F, Ghahremani MH, Aliebrahimi S, Shadboorestan A, Ostad SN (2019). Assessment of Cannabinoids Agonist and Antagonist in Invasion Potential of K562 Cancer Cells.. Iran Biomed J..

[38] Sadaria MR, Reppert AE, Yu JA, Meng X, Fullerton DA, Reece TB (2011). Statin therapy attenuates growth and malignant potential of human esophageal adenocarcinoma cells.. J Thorac Cardiovasc Surg..

[39] Kumar V, Dhanjal JK, Kaul SC, Wadhwa R, Sundar D (2021). Withanone and caffeic acid phenethyl ester are predicted to interact with main protease (M(pro)) of SARS-CoV-2 and inhibit its activity.. J Biomol Struct Dyn..

[40] Kumar V, Singh J, Hasnain SE, Sundar D (2021). Possible Link between Higher Transmissibility of Alpha, Kappa and Delta Variants of SARS-CoV-2 and Increased Structural Stability of Its Spike Protein and hACE2 Affinity.. Int J Mol Sci..

[41] Peach ML, Tan N, Choyke SJ, Giubellino A, Athauda G, Burke TR (2009). Directed discovery of agents targeting the Met tyrosine kinase domain by virtual screening.. J Med Chem..

[42] BIOVIA DS (2021). Discovery Studio Visualizer 4.5. San diego: Dassault systèmes..

[43] Damghani T, Sedghamiz T, Sharifi S, Pirhadi S (2020). Critical c-Met-inhibitor interactions resolved from molecular dynamics simulations of different c-Met complexes.. J Mol Struc..

[44] Wang L, Wang Y, Chen A, Teli M, Kondo R, Jalali A (2019). Pitavastatin slows tumor progression and alters urine-derived volatile organic compounds through the mevalonate pathway.. FASEB J..

[45] Kajstura M, Halicka HD, Pryjma J, Darzynkiewicz Z (2007). Discontinuous fragmentation of nuclear DNA during apoptosis revealed by discrete "sub-G1" peaks on DNA content histograms.. Cytometry A..

[46] Nagayama D, Saiki A, Shirai K (2021). The Anti-Cancer Effect of Pitavastatin May Be a Drug-Specific Effect: Subgroup Analysis of the TOHO-LIP Study.. Vasc Health Risk Manag..

[47] Kanugula AK, Dhople VM, Volker U, Ummanni R, Kotamraju S (2014). Fluvastatin mediated breast cancer cell death: a proteomic approach to identify differentially regulated proteins in MDA-MB-231 cells.. PLoS One..

[48] Jiang W, Hu JW, He XR, Jin WL, He XY (2021). Statins: a repurposed drug to fight cancer.. J Exp Clin Cancer Res..

[49] Sahebkar A, Kiaie N, Gorabi AM, Mannarino MR, Bianconi V, Jamialahmadi T (2021). A comprehensive review on the lipid and pleiotropic effects of pitavastatin.. Prog Lipid Res..

[50] Tjin EP, Groen RW, Vogelzang I, Derksen PW, Klok MD, Meijer HP (2006). Functional analysis of HGF/MET signaling and aberrant HGF-activator expression in diffuse large B-cell lymphoma.. Blood..

[51] van der Voort R, Taher TE, Derksen PW, Spaargaren M, van der Neut R, Pals ST (2000). The hepatocyte growth factor/Met pathway in development, tumorigenesis, and B-cell differentiation.. Adv Cancer Res..

[52] van der Voort R, Taher TE, Keehnen RM, Smit L, Groenink M, Pals ST (1997). Paracrine regulation of germinal center B cell adhesion through the c-met-hepatocyte growth factor/scatter factor pathway.. J Exp Med..

[53] Comoglio PM, Trusolino L, Boccaccio C (2018). Known and novel roles of the MET oncogene in cancer: a coherent approach to targeted therapy.. Nat Rev Cancer..

[54] Xu B, Muramatsu T, Inazawa J (2021). Suppression of MET Signaling Mediated by Pitavastatin and Capmatinib Inhibits Oral and Esophageal Cancer Cell Growth.. Mol Cancer Res..

