# Supplementary material for: Elucidating the Inhibitory Potential of Statins Against Oncogenic c-Met Tyrosine Kinase Through Computational and Cell-based Studies
Source: Iran J Pharm Res. 2025 Aug 16;24(1):e158845. doi: 10.5812/ijpr-158845 (PMC12523866; doi:10.5812/ijpr-158845)
Supplement: ijpr-24-1-158845-s001.pdf [file ijpr-24-1-158845-s001.pdf]

a b

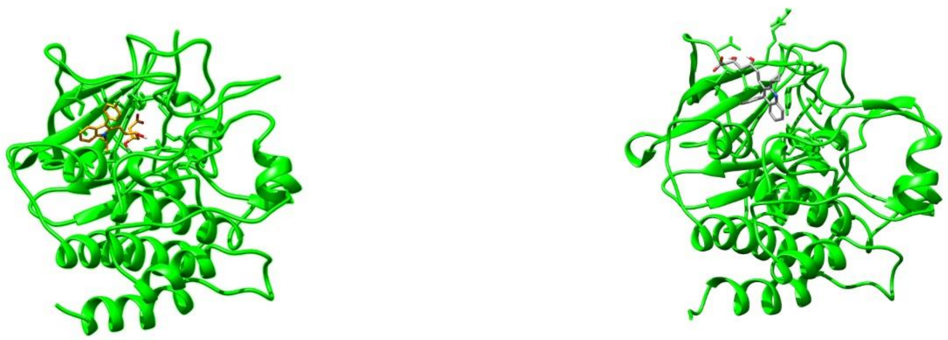

Appendix 1: MD simulation output represents the possible interacting model of protein-ligand complex three-dimensionally between c-Met receptor protein (PDB ID: 4xmo) (ribbon structure highlighted in green) active binding site and HMG-CoA reductase inhibitors, including (a) fluvastatin (highlighted in yellow) and (b) pitavastatin (highlighted in white), extracted at 25 ns of simulation.

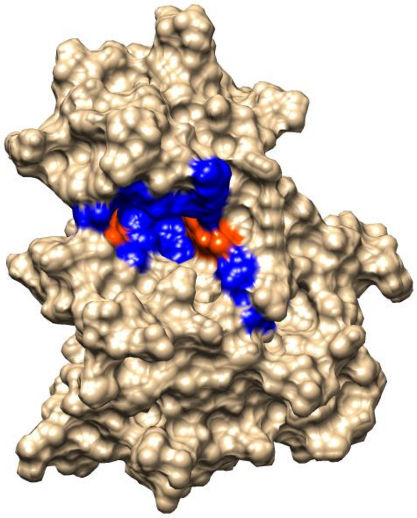

Appendix 2: 3D surface structure of c-Met receptor protein (PDB ID: 4xmo) (colored in white) representing the hydrophobic pocket (colored in blue) and the ATP binding site (colored in red). Hydrophobic pocket is consisting of D1222 R1227, A1226, G1085, L1140, A1221, D1228, D1231, Y1234, I1084, K1110, V1092, A1108, L1157, Y1159, G1163, N1209, P1158, R1086, and M1211 residues. The ATP binding site comprised of Y1230, M1160, and Y1159 residues.
